# Current Understanding and Future Research Direction for Estimating the Postmortem Interval: A Systematic Review

**DOI:** 10.3390/diagnostics15151954

**Published:** 2025-08-04

**Authors:** Gabriela Strete, Andreea Sălcudean, Adina-Alexandra Cozma, Carmen-Corina Radu

**Affiliations:** 1 Department of Psychiatry, “George Emil Palade” University of Medicine, Pharmacy, Science and Technology of Târgu Mureș, 540142 Târgu Mureș, Romania; elena.buicu@umfst.ro; 2 Department of Ethics and Social Sciences, “George Emil Palade” University of Medicine, Pharmacy, Science and Technology of Târgu Mureș, 540142 Târgu Mureș, Romania; andreea.salcudean@umfst.ro; 3 Department of Forensic Medicine, Satu Mare County Emergency Hospital, 440033 Satu Mare, Romania; 4 Department of Forensic Medicine, “George Emil Palade” University of Medicine, Pharmacy, Science and Technology of Târgu Mureș, 540142 Târgu Mureș, Romania; carmen.radu@umfst.ro

**Keywords:** postmortem interval, PMI, forensic entomology, RNA degradation, protein biomarkers, postmortem imaging, omics technologies, thanatomicrobiome, thanatological signs

## Abstract

**Background**: Accurate estimation of the postmortem interval (PMI) is critical in forensic death investigations. Traditional signs of death—algor mortis, livor mortis, and rigor mortis—are generally reliable only within the first two to three days after death, with their accuracy decreasing as decomposition progresses. This paper presents a systematic review conducted in accordance with PRISMA guidelines, aiming to evaluate and compare current methods for estimating the PMI. Specifically, the study identifies both traditional and modern techniques, analyzes their advantages, limitations, and applicable timeframes, critically synthesizes the literature, and highlights the importance of combining multiple approaches to improve accuracy. **Methods**: A systematic search was conducted in the PubMed, Scopus, and Web of Science databases, following the PRISMA guidelines. The review included original articles and reviews that evaluated PMI estimation methods (through thanatological signs, entomology, microbial succession, molecular, imaging, and omics approaches). Extracted data included study design, methodology, PMI range, and accuracy information. Out of the 1245 identified records, 50 studies met the inclusion criteria for qualitative synthesis. **Results**: Emerging methods, such as molecular markers, microbial succession, omics technologies, and advanced imaging show improved accuracy across extended postmortem intervals. RNA degradation methods demonstrated higher accuracy within the first 72 h, while entomology and microbial analysis are more applicable during intermediate and late decomposition stages. Although no single method is universally reliable, combining traditional and modern approaches tailored to case-specific factors improves overall PMI estimation accuracy. **Conclusions**: This study supports the use of an integrative, multidisciplinary, and evidence-based approach to improve time-since-death estimation. Such a strategy enhances forensic outcomes by enabling more precise PMI estimates in complex or delayed cases, increasing legal reliability, and supporting court-admissible expert testimony based on validated, multi-method protocols.

## 1. Introduction

Accurately determining the PMI is a fundamental objective in forensic pathology, with significant implications for medico-legal investigations, time-of-death estimation, and judicial proceedings.

Despite the long-standing interest in PMI estimation, no single method currently provides sufficient accuracy across all decomposition stages and environmental contexts. While traditional thanatological signs remain useful during the early postmortem period, their precision markedly declines beyond 48–72 h. In recent years, emerging techniques—including molecular, microbiological, and omics technologies, as well as imaging approaches—have shown promise. However, the existing literature is fragmented and often limited to isolated methodological perspectives, lacking a comprehensive synthesis.

This review aims to consolidate and critically evaluate both conventional and emerging PMI estimation approaches, addressing their specific windows of applicability, methodological limitations, and interdependencies. By systematically analyzing and comparing diverse methods—including classical signs, entomological data, biochemical markers, omics tools, and imaging technologies—this paper aims to guide forensic practitioners and researchers in selecting the most appropriate techniques for various case scenarios. Furthermore, by highlighting inconsistencies, research gaps, and experimental limitations, the review provides a roadmap for future validation efforts and the integration of interdisciplinary approaches.

The novelty of this work lies in its comprehensive scope, structured comparative analysis, and emphasis on multimodal combinations tailored to case-specific variables. This integrative perspective is crucial at a time when forensic science is increasingly demanding evidence-based, multidisciplinary approaches that are suitable for legal scrutiny.

Conventional methods for PMI estimation are based on the observation of early postmortem changes, particularly algor mortis (cooling of the body), livor mortis (postmortem hypostasis), and rigor mortis (stiffening of muscles). Although widely applied in practice, these methods are affected by numerous extrinsic (ambient temperature, humidity, clothing, body position) and intrinsic (age, body mass, health status) factors.

Forensic pathologists routinely assess these thanatological signs during the early stages of the postmortem examination [1]. Although the standard forensic literature often reports generalizations, such as body temperature decreasing by approximately 1 °C per hour during the first hours postmortem, livor mortis becoming fixed within 15–24 h, and rigor mortis beginning around 1–2 h, peaking at 14–24 h, and resolving after 24–36 h [2], these estimates are overly simplistic and potentially misleading [3,4]. Their interpretation requires careful correlation with environmental factors, scene findings, and autopsy observations. For example, the widely used Henssge nomogram provides a more accurate estimation of the postmortem interval, as it accounts for variables, such as ambient temperature, body mass, and correction factors [5]. The recent literature has increasingly criticized the use of fixed rates, such as the 1 °C per hour cooling rule, for oversimplifying the dynamic and nonlinear nature of postmortem temperature loss [4,5].

Supravital reactions, including electrical stimulation of muscles, mechanical stimuli, or pharmacological tests on the iris, can sometimes support PMI estimation in the very early stages postmortem. However, their utility is limited to a narrow time window and must not be confused with biochemical analyses. In contrast, the analysis of vitreous humor potassium concentration, as well as gastric evaluation, provides biochemical means to estimate PMI over a slightly longer timeframe [6,7,8,9]. While valuable, both are subject to significant biological variability and require standardization.

Forensic entomology is a well-established tool for estimating the PMI, particularly in late stages of decomposition. It relies on the identification of insect species present on the body, their developmental stages, and succession patterns. Although environmental factors can affect colonization timing, entomological evidence remains one of the most reliable methods for long-term PMI estimation [10] and must be interpreted by trained experts.

Similarly, forensic radiology—though less standardized—has demonstrated potential through imaging signs of decomposition, particularly in indoor deaths and hospital settings.

Imaging and sensor-based approaches offer non-invasive techniques for PMI estimation. Advanced imaging methods, including infrared thermography [11], PMCT (including pilot animal studies showing organ-specific CT changes correlated with PMI) [12,13,14,15], and MRI [16], have been studied for early PMI estimation, but no standardized imaging tool currently exists for PMI determination.

Recent studies have investigated RNA degradation as a molecular approach for PMI estimation [17,18], although challenges related to sample integrity and standardization still remain. Further discussion of their applicability is provided in the relevant section.

In addition, thanatomicrobiome studies, analyzing the dynamic microbial communities that evolve in and on the body after death, have revealed potential for PMI estimation through microbial succession models. Nonetheless, most studies remain preliminary or animal-based, with human applications still under development. Their predictive capacity is promising but far from validated for routine forensic practice [19].

Omics technologies (untargeted proteomics, metabolomics, and lipidomics) are emerging as holistic approaches to PMI [20]. These techniques offer novel insights into decomposition at the molecular level. However, they remain primarily research tools, requiring further validation, larger datasets, and the development of mathematical models to minimize variability and error margins [21].

Additionally, decomposition scoring systems, such as the total body score (TBS), and accumulated degree days (ADD), provide objective frameworks to quantify decomposition progress in outdoor or buried cases [22]. These methods support PMI estimation over extended intervals and complement other forensic findings.

The estimation of the time elapsed between death and the discovery of the body remains a major challenge in forensic science and criminal investigations [23,24,25,26]. Despite all the methods proposed over the years, there is a clear need for systematic studies that critically evaluate and compare these techniques across real-world contexts and postmortem intervals.

This systematic review, conducted in accordance with PRISMA guidelines, aims to provide a structured, comparative analysis of both traditional and emerging PMI estimation methods. We focus on their accuracy, applicability, time-specific reliability, and practical integration in forensic workflows.

By analyzing conventional thanatological signs alongside entomological, microbiological, molecular, imaging, and omics-based approaches, this review offers guidance for forensic practitioners in selecting context-appropriate techniques. Furthermore, we identify key research gaps and propose future directions for the development of standardized, evidence-based protocols.

## 2. Materials and Methods

### 2.1. Literature Search Strategy

This systematic review was conducted in accordance with the PRISMA guidelines [27]. A structured literature search was performed using three major electronic databases, namely PubMed, Scopus, and Web of Science. These databases were selected to ensure comprehensive coverage of the biomedical, forensic, and life science literature relevant to PMI estimation.

The search strategy included combinations of keywords and controlled vocabulary terms, such as “postmortem interval”, “PMI”, “thanatological signs”, “forensic entomology”, “RNA degradation”, “protein biomarkers”, “postmortem imaging”, “thanatomicrobiome”, and “omics technologies”.

No date restrictions were applied, although this systematic review aimed to identify and evaluate validated methods for estimating the PMI, with a particular focus on developments reported in the last 10 to 15 years. All selected papers were required to be written in English.

We restricted our evaluation to peer-reviewed journal articles, including both original research and review papers, that presented or assessed methods for PMI estimation.

### 2.2. Eligibility Criteria

Eligible studies met the following inclusion criteria:

- Original research articles or reviews focused on methods used to estimate the PMI;
- Inclusion of data on the accuracy, performance, feasibility, or applicability of these methods;
- Publication in a peer-reviewed scientific journal.

Both human- and animal-based studies were considered, as well as laboratory-based or field studies. Review articles were eligible if they synthesized empirical data or provided comparative evaluations of PMI estimation approaches.

The exclusion criteria were as follows:

- Studies not addressing PMI estimation as a primary objective;
- Papers focusing solely on extended PMIs outside the forensic relevance window;
- Articles lacking original data;
- Non-English publications;
- Duplicated datasets or overlapping publications from the same research group.

### 2.3. Study Selection

All search results were managed using Mendeley (v1.19.8) as a reference manager to compile all database search results. A total of 1245 records were identified from databases using key words/their combination. After merging the results and removing 245 duplicates, an additional 15 records were excluded through automated filters, and 20 were removed due to technical errors or incomplete metadata. This process resulted in 965 records screened by title and abstract.

**Figure 1:**
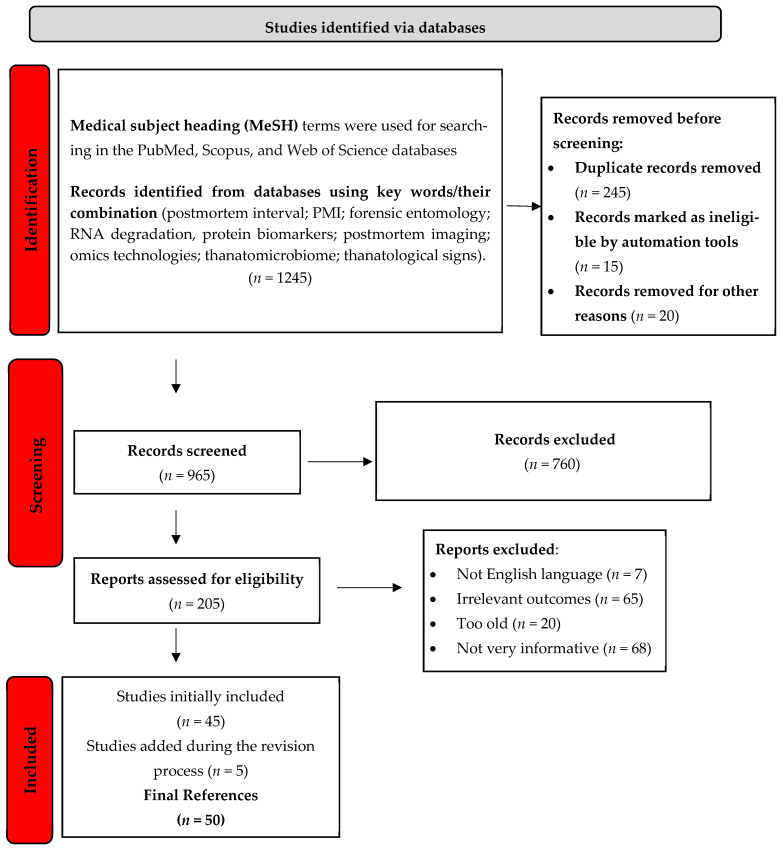
PRISMA flow diagram for the systematic review (the study selection process). This diagram highlights the number of records identified, screened, assessed, and the final studies included (*n* = 50).

Two authors independently screened the titles and abstracts for relevance to the study objectives. Discrepancies were resolved through discussion and consensus, with involvement of a third author when necessary. A total of 205 full-text articles were retrieved and assessed. Of these, 155 were excluded based on the following reasons: not in English (*n* = 7), irrelevant outcomes (*n* = 65), too old (*n* = 20), or not very informative (*n* = 68). Ultimately, 50 studies met all the eligibility criteria and were included in the qualitative synthesis. The study selection process is detailed in the PRISMA flow diagram (Figure 1).

### 2.4. Data Extraction and Synthesis

A standardized data extraction form was used to collect key information from each included study. The following data were extracted: study type (original vs. review, human vs. animal model), methodological approach (e.g., forensic entomology, molecular markers), sampling details, number of samples or cases analyzed, PMI range, and reported accuracy, correlation, or error measures.

Studies were categorized into the following methodological groups: (1) thanatological signs; (2) vitreous humor chemistry; (3) forensic entomology; (4) microbial succession (thanatomicrobiome); (5) molecular approaches (RNA-, DNA-, and protein-based); (6) imaging techniques; and (7) omics technologies (e.g., proteomics, metabolomics, lipidomics).

Due to the methodological heterogeneity across studies, a meta-analysis could not be conducted. Therefore, a qualitative synthesis was performed, highlighting the strengths, limitations, and applicable postmortem timeframes for each method. Statistical indicators, including estimation error and correlation coefficients, were presented when reported in the original studies.

No formal risk-of-bias assessment was applied due to the diversity of study designs and outcomes.

This review also aims to provide a replicable structure for future research, offering a clear methodological framework to facilitate comparison, validation, and the development of standardized approaches for PMI estimation in forensic practice.

In Figure 1, we illustrate the process of the study selection using a PRISMA flow diagram.

Standardized forms were used to collect essential data and findings from the 50 included studies, which were then analyzed and synthesized in this review. The extraction of data from experimental studies included study design elements (human case analysis vs. animal model, laboratory vs. field study) and method(s) evaluation (tested marker or technology), sample characteristics (number of bodies or samples and human or animal origin), PMI range examined, and estimated accuracy or error rates. We recorded the extent of the method coverage and primary findings about each approach in the review articles. The studies were organized into PMI estimation method categories for easier comparison.

The literature analysis produced seven main categories, which included (1) thanatological signs with algor, livor, and rigor mortis; (2) biochemical changes in vitreous humor chemistry; (3) forensic entomology (insect evidence); (4) microbial succession (thanatomicrobiome); (5) molecular methods (subdivided into RNA-based, DNA-based, and protein-based approaches); (6) imaging techniques, and (7) omics technologies (untargeted proteomics, metabolomics, etc.). We analyzed the findings by category while documenting the usual postmortem period for each method and the reported benefits and drawbacks. A summary table was developed based on the literature review to highlight the main features of each method (Table 1 in Section 3).

Due to the heterogeneity of methods and outcomes, a meta-analysis was not feasible; therefore, we conducted a qualitative systematic analysis. A risk-of-bias tool was not used formally because the studies employed different research designs. The goal of our data synthesis and interpretation was to help forensic practitioners understand the existing PMI estimation methods.

## 3. Results

A total of 50 studies met the inclusion criteria and were included in the qualitative synthesis. These studies explored a wide range of methodologies for estimating the PMI, from conventional thanatological signs to advanced molecular and imaging techniques. The results are presented in two parts, as follows:

- Table 1 provides a comparative synthesis of the main PMI estimation methods, including their principles, postmortem interval ranges, advantages, limitations, and key references.
- Table 2 presents detailed characteristics of each of the 50 included studies, outlining the methods, PMI range, advantages, and limitations.

The PRISMA flowchart (Figure 1) outlines the selection process.

The included studies were grouped into the following seven methodological categories based on the approach to PMI estimation: (1) thanatological signs; (2) vitreous humor chemistry; (3) forensic entomology; (4) microbial succession (thanatomicrobiome); (5) molecular approaches (RNA-, DNA-, and protein-based); (6) imaging techniques; and (7) omics technologies.

**Table 1:** Comparative summary of methods for PMI estimation, as reported in the literature.

Method	Principle and Markers	Typical PMI Range	Advantages	Limitations	Key References
1. Thanatological signs (algor, livor, and rigor mortis)	Physical postmortem changes: algor mortis (body cooling), livor mortis (postmortem hypostasis), and rigor mortis (muscle stiffening).	Immediate to ~2–3 days postmortem (early PMI)	Simple, quick, no special equipment; well-understood timeline in early phase.	Large variability due to ambient conditions (temperature, clothing, etc.) and body factors; not usable beyond ~72 h	[1,2,4,5,6]
2. Vitreous humor chemistry	Rise in electrolyte levels (e.g., potassium, hypoxanthine) in the eye fluid after death.	1–10 days postmortem (early-to-mid PMI)	Provides semi-quantitative estimates up to ~1 week; relatively independent of external factors (vitreous is protected).	Requires lab analysis; influenced by temperature and certain pathologies; less accurate as decomposition advances	[6]
3. Forensic entomology	Insect colonization and life cycle stages on the corpse (e.g., blowfly maggot development, insect succession patterns).	Days to months postmortem (middle-to-late PMI)	Can yield PMI estimates long after death when insects have had time to colonize; well-established developmental timelines for many species.	Dependent on environmental conditions (temperature, season) and insect access to body; requires entomological expertise; PMI estimates are given as ranges	[10]
4. Microbial succession	Changes in bacterial and fungal community composition over time (detected via 16S rRNA gene sequencing or other genomic tools).	Days to weeks postmortem (middle PMI)	Potential “microbial clock” offers objective biochemical marker; microbes present in all bodies and environments; not reliant on insect presence.	Still experimental—high variability between individuals and environments; influenced by soil, temperature, burial; requires advanced DNA sequencing and bioinformatics; not yet standardized	[28]
5. Molecular methods RNA-based methods DNA-based methods Protein-based methods	Degradation of mRNA transcripts and/or changes in gene expression postmortem (e.g., loss of RNA integrity, or differential expression of certain genes over time).	Hours to a few days postmortem (early PMI)	Quantifiable molecular changes; tissue-specific RNA markers can improve short-term PMI precision; useful when physical signs are equivocal.	RNA degrades rapidly; sensitive to temperature and pH; requires prompt sample collection and specialized lab equipment (RT-qPCR, sequencing); significant tissue specificity	[17,29]
	Degradation of genomic DNA or DNA fragmentation patterns	Hours to days	DNA is more stable than RNA; some studies report a correlation between fragmentation and PMI; applicable to many tissue types.	High variability; influenced by storage, cause of death, environmental conditions; still in experimental stages	[30]
	Time-dependent proteolysis and modification of proteins in tissues (e.g., appearance of protein fragments, loss of specific proteins, proteomic profile changes).	Days to weeks postmortem (early-to-mid PMI)	Proteins are more stable than RNA, enabling longer detection; many tissues (muscle, bone, etc.) show measurable protein degradation timelines; amenable to mass spectrometry.	Requires laboratory analysis; some interindividual variation in protein levels; need for large postmortem protein databases; mostly in research phase	[18]
6. Imaging techniques	Postmortem imaging or sensing (e.g., infrared thermography for cooling, PMCT for gas/fluid distribution, MRI for tissue changes).	Minutes to hours (thermal), up to days (selected imaging signs)	Non-invasive, can be performed when autopsy is not possible; thermal imaging provides continuous data on cooling; CT/MRI can document internal changes without dissection.	Thermal methods limited to very early PMI; CT/MRI changes are not specific or quantitative for time since death; interpretation requires expertise; equipment not always available in forensic units.	[11,12,13,14,15,16]
7. Omics technologies (proteomics, metabolomics, and lipidomics)	High-throughput analysis of a broad array of biomolecules to identify signature changes (e.g., protein profile shifts, metabolite accumulation) over time.	Hours to ~2 months (short-to-mid PMI)	Holistic approach—can discover novel biomarkers; data-driven models may increase accuracy; metabolomics can capture chemical changes during decomposition.	Requires sophisticated instrumentation and bioinformatics; large datasets needed to find robust markers; many studies are on animal models; current focus mostly on <7 day PMI	[20]

The postmortem interval ranges, where each method excels, are demonstrated in Table 1.

**Table 2:** Synoptic summary of the 50 studies included in the systematic review on PMI estimation methods.

Study	Method	PMI Range	Advantages	Limitations
1. Franceschetti et al., 2023 [6]	Literature review of various PMI estimation techniques	Late PMI	Comprehensive overview	Does not present new data
2. Laplace et al., 2021 [29]	Comparative cooling methods	0–36 h	Method comparison for early PMI	Precision varies by method
3. Henssge & Madea, 2007 [5]	Temperature-based PMI estimation	0–36 h	Empirical formulas	Environmental factors crucial
4. Singh et al., 2025 [3]	Comprehensive review of PMI estimation methods	General (from immediate to late PMI)	Summarizes traditional and emerging techniques; highlights recent advances and challenges in a forensic context	No new experimental data; narrative synthesis; broad rather than focused on a single method.
5. Sturner & Gantner, 1964 [24]	Potassium in vitreous humor	0–120 h	Historical foundation	Outdated sample control
6. Lange et al., 1994 [8]	Vitreous potassium across studies	0–100 h	Meta-analytic insight	Interstudy variability
7. Zilg et al., 2015 [7]	Vitreous potassium corrected for age/temp	0–100 h	Validated mathematical model	Sampling precision needed
8. McCleskey et al., 2016 [9]	Review of vitreous humor methods	0–120 h	Integrative synthesis of existing analytical models	Secondary analysis without primary data
9. Cordeiro et al., 2019 [23]	Vitreous humor biochemistry, temp, body weight	0–120 h	Combines multiple indicators	Affected by environmental factors
10. Madea & Rödig, 2006 [1]	Excitability testing	0–12 h	Immediate response	Limited to early PMI
11. Bansode et al., 2025 [31]	Forensic entomology-simulation tools and entomotoxicology	Several days to weeks	Covers modern predictive models, role of necrophagous species beyond dipterans, drug/toxin effects, environmental factors; practical forensic recommendations	Narrative review; no experimental data; applicability may vary by geography
12. Schoenly et al., 1992 [32]	Algorithm from arthropod succession	0–15 days	Systematic entomological model	Species- and region-dependent
13. Schoenly et al., 1996 [33]	Statistical modelling of insect succession	Several days to weeks	Quantitative estimation	High variability and uncertainty in some cases
14. Marchenko, 2001 [34]	Cadaver associated entomofauna	Several days to weeks	Taxonomic diversity data	Geographic dependency
15. Donovan, 2006 [35]	Larval growth data	Up to approx. 30 days	Empirical developmental data with temperature-dependent ADH model	Laboratory conditions may not fully reflect environmental variability
16. Michaud & Moreau, 2009 [36]	Insect visitation prediction by degree day	0–10 days	Temperature-adjusted	Needs accurate weather data
17. Reibe et al., 2010 [37]	Simulation model for Lucilia sericata	0–15 days	Standardized entological tool	Simulation assumptions
18. Matuszewski, 2011 [38]	Pre-appearance interval from temperature	Early PMI	Quantitative model	Species specific
19. Mohr & Tomberlin, 2015 [39]	Adult blowfly attendance model	1–7 days	Specific to dipteran behavior	Not generalizable to all insects
20. Matuszewski, 2017 [40]	Qualitative indicator integration	0–10 days	Holistic approach	Subjective indicator selection
21. Matuszewski & Fratczak, 2018 [41]	PMI based on adult size at emergence in Creophilus maxillosus	7–30 days	Improves accuracy in insect age estimation	Requires larval rearing to adulthood; limited to one beetle species
22. Matuszewski, 2021 [10]	Forensic entomology	Early-to-mid PMI	Species-specific PMI estimation	Environmental dependency and local fauna
23. Obafunwa et al., 2025 [42]	Forensic entomology	Several days to weeks	Established method	Species and environment dependent
24. Metcalf et al., 2013 [19]	Microbial clock (mouse model)	0–30 days	High correlation with time	Animal model
25. Bell et al., 2018 [43]	Thanatomicrobiome sex-based differences	0–10 days	Sex-specific microbial patterns	Requires sequencing
26. Lutz et al., 2020 [44]	Microbiome shifts in internal organs	0–30 days	Internal organ focus	Environmental sensitivity
27. Tozzo et al., 2022 [28]	Microbiome analysis via 16s rRNA	0–15 days	Sensitive and specific	Requires sequencing infrastructure
28. Zapico & Adserias-Garriga, 2022 [45]	Human tissue and microbiome shifts	0–20 days	Human-based, multiple matrices	Sample variability
29. Wang et al., 2022 [46]	AI-based microbiome analysis	0–10 days	Automation, scalability	Complexity, data training needed
30. Pittner et al., 2016 [47]	Muscle protein degradation	0–240 h	Time-resolved data	Affected by storage conditions
31. Zhu et al., 2017 [17]	Gene expression analysis	0–48 h	Quantifiable molecular changes	Affected by RNA degradation
32. Prieto-Bonete et al., 2019 [48]	Bone protein profile	Late PMI	Durability of sample	Bone preservation required
33. Zissler et al., 2020 [18]	Protein degradation	0–10 days	Biological consistency	Requires lab processing
34. Javan et al., 2020 [49]	Thanatotranscriptome in liver	0–48 h	Gene-based marker approach	Requires RNA integrity
35. Cianci V., et al., 2024 [21]	MicroRNA analysis	0–5 days	Increased RNA stability; potential for precise early PMI estimation	Experimental; temperature-sensitive; lacks standardization
36. Gerra M.C., et al., 2024 [50]	Epigenetic markers	0–10 days	Potentially stable and specific postmortem markers	Highly experimental; requires validation and standardization
37. Bhoyar L., et al., 2025 [30]	DNA degradation via comet assay bone imaging	0–5 days	Sensitive detection of DNA degradation; relatively accessible technique	Experimental; needs validation in human forensic context
38. Wang et al., 2017 [12]	CT imaging	0–7 days	Structural visualization	High cost, accessibility
39. Chan et al., 2021 [11]	Infrared thermography	0–24 h	Non-invasive, real-time	Sensitive to environment
40. Schmidt et al., 2022 [51]	Handheld NIR spectroscopy on bone	Weeks to years	Portable and fast	Surface degradation effects
41. Klontzas et al., 2023 [13]	CT radiomics	0–72 h	Automated analysis	Requires imaging and software
42. Shen et al., 2024 [14]	Postmortem CT (animal study)	0–96 h	Multi-tissue analysis	Animal model; translation to humans uncertain
43. Procopio et al., 2018 [52]	Forensic proteomics in bones	Advanced decomposition	Applicable to skeletonized remains	Specialized techniques required
44. De-Giorgio et al., 2025 [15]	Radiomic analysis of brain ventricles (CT)	0–5 days	Quantitative imaging data	Needs CT and postprocessing
45. Du et al., 2018 [53]	Muscle metabolic profiling (rat)	0–96 h	Distinct metabolic signatures	Animal model
46. Ferreira et al., 2018 [54]	Transcriptome analysis postmortem	0–48 h	Molecular resolution	Affected by ischemia and degradation
47. Choi et al., 2019 [55]	Proteomics for PMI biomarkers	0–10 days	Discovery of new biomarkers	Requires MS equipment
48. Bonicelli et al., 2022 [56]	ForensOMICS (multi-omics in bone)	Late PMI	Integrated data layers	Complex interpretation
49. Li et al., 2024 [57]	Multi-omics integration	0–10 days	High-resolution molecular insight	Requires bioinformatics expertise
50. Secco et al., 2025 [20]	Multi-omics approach	0–10 days	Comprehensive data integration	Complex and resource-intensive

Table 2 provides details of the 50 included studies, with information on method, PMI range, advantages, and limitations.

The fifty studies selected for this review span a publication range from 2001 to 2025, with the vast majority (80%) published within last 10–15 years, reflecting the increasing interest in novel PMI estimation approaches. Earlier studies, such as Sturner & Gantner (1964) [24], Lange et al. (1994) [8], and Schoenly (1992, 1996) [32,33], were included due to their foundational or historical relevance in the forensic literature. For example, although it was published in 1964, the study by Sturner and Gantner was included for its significance in establishing the forensic use of vitreous potassium levels, which formed the basis for many subsequent models [31].

The thanatological signs of algor, livor, and rigor mortis are primarily observable within the first two-to-three days postmortem [6]. Although these indicators are commonly assessed during autopsy or at the death scene, their evaluation is not always straightforward. Factors, such as immersion in water, hypothermia, or environmental variability, can compromise the reliability of algor mortis, especially when using the Henssge nomogram. Rigor mortis can be confused with cold stiffening or cadaveric spasm, which may lead to misinterpretation. As Henssge and Madea (2007) note, accurate algor mortis-based PMI estimation requires strict control of environmental and body parameters [5].

Vitreous humor biochemistry, especially potassium levels, extended the estimation window up to 120 h, though with variable precision and dependence on temperature correction factors [7].

According to Matuszewski (2021), forensic entomology remains a powerful method for PMI estimation; however, it faces ongoing challenges due to environmental variability and the influence of drugs or toxins on insect development [10]. Entomological estimates require adjustments under variable condition, such as delayed maggot development in cold environments or limited insect access in indoor settings and may become entirely unreliable under certain circumstances.

Microbial succession also falls within the intermediate postmortem range. Tozzo et al. (2022) [28] reviewed studies demonstrating that “microbial clocks” can estimate the PMI from several days to several weeks, with specific bacterial species correlating with different stages of decomposition. The review highlighted the need for further human-based studies to account for variability in individual microbiomes, cause of death, and such factors as body enclosure or conditions [28].

Postmortem RNA and protein degradation show distinct temporal patterns during the early-to-mid period after death. While muscle and brain tissues often exhibit rapid fragmentation and expression changes in the first 12–48 h postmortem, Zapico and Adserias-Garriga (2022) demonstrate that other human tissues—including liver, lung, cardiac blood, skeletal muscle, and bone—have also been analyzed with transcriptomic and proteomic methods, confirming tissue-specific degradation dynamics and supporting the broader applicability of RNA-based biomarkers for PMI estimation [45]. The application of RNA-based methods becomes particularly useful when examining decomposing bodies beyond the timeframe in which algor or rigor mortis offer reliable information. These molecular techniques may serve as valuable complements to traditional PMI estimation methods, especially when physical indicators begin to lose precision. Although RNA analysis has not yet been fully validated for routine forensic use, it may still provide valuable supplementary data in the early postmortem period and refine time-of-death estimates when used alongside conventional markers.

Research by Zhu et al. (2017) demonstrated that extensive gene expression changes occur in various tissues in relation to the time since death, suggesting the potential for developing mRNA-based markers to estimate the PMI [17].

Protein-based analyses have shown applicability over longer postmortem periods than RNA-based approaches. The degradation rates of structural muscle proteins and enzymes allow detection for several days to weeks, and in some cases, proteins may remain detectable in bones or mummified tissues even after extended intervals.

The systematic review conducted by Zissler et al. (2020) analyzed multiple studies on protein degradation and concluded that the observed degradation patterns are sufficiently consistent to support potential future use in forensic [18]. The authors highlighted that troponin and tropomyosin-proteins involved in muscle contraction-undergo identifiable postmortem breakdown, making them suitable markers for estimating the time since death.

The imaging techniques included in Table 1 are included primarily for context, as the final pool of selected studies did not focus exclusively on imaging-based research. Infrared thermography has occasionally been employed at crime scenes to measure heat dissipation from the body, assisting in the estimation of algor mortis.

PMCT has demonstrated its ability to visualize gas accumulation in tissues and blood vessels, resulting from bacterial decomposition. Research in forensic radiology shows that CT imaging can help stage decomposition by quantifying gas presence in organs, such as the liver and heart, providing indirect indicators of the PMI. However, decomposition rates are highly variable, which limits precise time-of-death estimation using imaging alone. Thus, imaging serves best as a complementary method—particularly in cases beyond the early PMI—where significant internal gas and fluid changes suggest longer PMIs [12].

As forensic science evolves, the field continues to move beyond structural assessments, like imaging, towards integrative molecular profiling.

Omics technologies represent the current state-of-the-art in PMI estimation. These methods analyze broad patterns across large-scale biological datasets rather than relying on individual markers. For example, proteomic profiling can detect combinations of protein fragments whose abundance patterns distinguish between a 3-day-old and a 10-day-old corpse. Similarly, metabolomic profiling identifies characteristic patterns of small molecules—such as short-chain fatty acids, amino acids, and purines—that collectively serve as a “molecular fingerprint” of specific decomposition stages [20].

The study by Du et al. (2018) exemplifies this approach by demonstrating significant changes in metabolite concentrations in rat muscle tissues within the first 3 days postmortem. These data were then processed by statistical models to accurately classify samples based on PMI [53].

According to Secco et al. (2025), most existing omics studies have been conducted on animal models and are limited to short postmortem intervals [20]. The authors emphasize the need for human-based research and the investigation of longer PMIs. Omics data analysis relies on sophisticated computational tools, including machine learning algorithms, to identify and interpret complex patterns. These methods require extensive training datasets and may risk overfitting to specific conditions.

Overall, the findings of this systematic review confirm that no single method is universally applicable across all PMIs. Each approach contributes valuable insights within specific time windows and under particular conditions. Conventional methods remain useful during the early postmortem period but lose reliability as decomposition progresses [6].

Forensic entomology and microbial succession methods covered longer intervals, from several days to weeks. Entomology remains the most validated late-stage method, while microbial and omics approaches are still largely experimental, especially in human models. Molecular markers, particularly RNA and protein degradation, demonstrated promise within 0–10 days, though most evidence comes from controlled animal studies. RNA integrity is useful for short PMIs (<48 h), while protein-based approaches have a broader window (up to 2 weeks). Imaging methods, such as infrared thermography and PMCT, have the potential for organ-specific decomposition tracking.

The results of the included studies demonstrate that each method has specific strengths-such as ease of use, proven accuracy whithin certain contexts, or potential for future innovation-as well as corresponding limitations, including sensitivity to environmental conditions, reliance on specialized analytical tools, or being in early developmental stages. These aspects are further explored in the Discussion section.

### Risk of Bias and Limitations

This systematic review included studies with heterogeneous methodologies, target populations (including both human and animal models), and analytical approaches, which limits direct comparability and precludes the possibility of conducting a quantitative meta-analysis. Although the inclusion strategy was revised, a formal risk-of-bias assessment tool was not applied due to the methodological diversity of the included studies. Consequently, potential sources of bias—such as sample selection, reporting inconsistencies, or methodological variation—may be present. Despite these limitations, the qualitative synthesis was designed to offer a balanced and comprehensive overview of current methodologies for estimating the PMI.

## 4. Discussion

### 4.1. General Overview

Estimating the PMI remains a critical yet complex challenge in forensic medicine. Over the past decades, numerous methodologies have emerged to refine time-of-death estimation, ranging from classical physical signs to molecular- and omics-based innovations. Traditional methods—such as thanatological changes and vitreous humor chemistry—continue to serve as the foundation of early PMI assessment due to their accessibility and rapid applicability. However, their accuracy is often compromised by environmental variability and individual differences, leading to increased interest in objective and quantifiable biomarkers.

Recent advances in forensic science have introduced innovative approaches, such as RNA degradation, proteomics, metabolomics, microbiome analysis, and postmortem imaging. These techniques aim to reduce subjectivity, improve reproducibility, and expand the PMI estimation window. Despite promising preliminary results, many of these methods remain in the experimental phase and lack standardization for routine forensic use.

Systematic reviews, such as those by Singh et al. (2025) and López-Lázaro & Castillo-Alonso (2024), emphasize the need for multi-parametric, integrative approaches that combine traditional and modern tools to improve reliability and admissibility in legal contexts [3,58]. Furthermore, the literature highlights a shift towards computational modeling, the use of artificial intelligence, and the development of standardized protocols for postmortem sampling and biomarker analysis.

This review categorized the included studies into seven methodological groups—thanatological signs, vitreous humor chemistry, forensic entomology, microbial succession, molecular techniques (RNA, proteins, and DNA), imaging modalities, and omics technologies—each with distinct advantages and limitations in estimating PMI. The following sections present a critical discussion of each category, integrating the recent literature, methodological progress, and current limitations.

### 4.2. Thanatological Signs (Algor, Livor, and Rigor Mortis)

Traditional thanatological signs remain among the most accessible and widely used indicators for early PMI estimation. These methods are frequently employed due to their simplicity, no-cost application, and feasibility in a broad range of forensic settings, particularly in the first hours after death. In a comparative insight study, Madea and Rödig (2006) demonstrated the utility of algor and rigor mortis under standardized conditions, achieving acceptable accuracy within the first 24–48 h postmortem [1]. In contrast, Zhu et al. (2017) observed that in hot and humid environments, the reliability of these signs decreases rapidly, emphasizing the need for complementary molecular approaches [17].

Algor mortis is affected by a range of external and intrinsic variables, including ambient temperature, clothing, body mass, and humidity. While nomograms, such as Henssge’s, have attempted to systematize this process and provide estimations with defined error margins and confidence intervals, precision remains limited under fluctuating environmental conditions. The Henssge nomogram, in particular, provides PMI estimates within a 95% confidence interval, under standardized conditions—a fact that must be more clearly acknowledged in PMI estimation protocols [5].

Rigor mortis develops in sequence with livor mortis, though its onset and progression can vary depending on physical activity prior to death (e.g., struggle or exercise), as well as environmental influences that may delay or extend its duration. It is important to note that algor mortis estimation using the Henssge nomogram can be unreliable in hypothermia or aquatic deaths, and rigor mortis may be confused with cold stiffening.

Recent studies, including those by Singh et al. (2025), emphasize that while these methods provide valuable initial orientation, they should no longer be viewed as standalone indicators of PMI [3]. Instead, they serve best when integrated with complementary methods, such as vitreous chemistry or entomological analysis, to improve reliability and reduce the risk of misinterpretation.

### 4.3. Biochemical Techniques

The analysis of vitreous humor has long been considered one of the most reliable biochemical approaches for early PMI estimation, particularly during the first 120 h postmortem [7,8,9,23,24].

Key markers studied in vitreous humor include potassium, hypoxanthine, urea, and creatinine. Among these, potassium concentration shows a well-documented postmortem increase due to cell membrane breakdown, allowing for the application of regression models to estimate the PMI. Zilg et al. (2015) proposed a mathematical model that accounts for individual variation by correcting for age and ambient temperature, improving the accuracy of potassium-based estimates [7].

However, as highlighted by López-Lázaro and Castillo-Alonso (2024) in their recent systematic review, significant limitations remain. These include interindividual variability, pathological states influencing baseline electrolyte levels, and the lack of universal calibration for estimation formulas [58]. Moreover, the utility of vitreous markers sharply declines after decomposition begins, especially beyond 5 days postmortem.

While vitreous humor analysis remains an essential adjunct in forensic practice, current evidence suggests that it is best employed in combination with physical signs or other biochemical and molecular markers to enhance reliability across different postmortem windows.

### 4.4. Forensic Entomology

Forensic entomology is a well-established discipline used in the estimation of the postmortem interval, particularly in the intermediate to late stages of decomposition (from several days to weeks or months postmortem). According to Matuszewski S. (2021), it is based on the predictable colonization patterns of necrophagous insects, primarily blowflies (Calliphoridae), but also includes other taxa, such as flesh flies (Sarcophagidae) and beetles (e.g., Dermestidae, Silphidae) [59].

Forensic entomologists have developed several methods for estimating the PMI, based either on insect developmental stages [10,34,60] or on faunal succession patterns [32,33,38].

Classical methods rely on the analysis of insect developmental stages, especially maggot age, to estimate the minimum PMI. However, as highlighted by Bansode et al. (2025), modern forensic entomology has evolved to incorporate simulation models, climate-adjusted degree-day calculations, and the use of software tools for species-specific growth rates [31]. These advancements enhance the precision of PMI estimates and can be adapted for varying environmental contexts.

Despite its strengths, entomology-based PMI estimation faces several limitations. Insect development is highly sensitive to temperature, humidity, seasonality, and even body accessibility. For example, according to Amendth et al. (2007), delayed colonization may occur in indoor deaths or in covered or wrapped bodies, which can significantly alter the PMI calculation [61]. Moreover, Bansode et al. (2025) emphasized that exogenous substances, such as drugs, toxins, and chemical residues can influence larval development rates, adding further complexity [31].

Modern forensic entomology has begun incorporating predictive simulation models that can account for fluctuating temperatures and microclimatic variability, with the aim of improving the accuracy of PMI estimations in diverse environmental contexts [62].

An additional challenge is the taxonomic identification of species, which requires expert knowledge, particularly in geographic regions where species diversity is high and reference growth data may be lacking. Studies have also shown that overreliance on Calliphoridae alone may lead to inaccuracies; alternative decomposers, such as beetles, should be integrated into routine entomological assessments, especially for extended PMI cases [31].

Overall, forensic entomology remains indispensable in PMI estimation when decomposition is advanced and other methods become unreliable. However, its effectiveness depends heavily on standardized protocols, entomological expertise, and integration with other forensic findings.

### 4.5. Microbial Succession (Thanatomicrobiome)

The study of microbial succession, often referred to as the “microbial clock” has gained considerable attention in recent years as a promising molecular approach for estimating PMI, especially during the intermediate postmortem period (from a few days to several weeks). This method relies on predictable changes in the composition and abundance of bacterial and fungal taxa in and on the body following death [63].

Several studies, such as Metcalf et al. (2013) [19], laid the groundwork using animal models, particularly mice, to demonstrate that microbial communities shift in a temporally structured manner after death. However, these findings have not yet been fully replicated in human-based studies, limiting their forensic applicability [19].

According to Tozzo et al. (2022) [28] DNA-based tools, like 16S rRNA sequencing, enable the identification of microbial taxa associated with specific decomposition phases. For example, Clostridia species have been linked to distinct postmortem intervals in human cadavers [28].

More recent reviews, including those by Tozzo et al. (2022) and Cianci et al. (2024), emphasize that although microbial succession shows potential for developing quantitative PMI models, the method remains experimental and non-standardized [21,28].

The variability observed across studies on the thanatomicrobiome arises from multiple confounding factors, including host-specific variables (genetics, lifestyle cause of death, and health status); environmental conditions such as soil composition, temperature, humidity, insect activity, and burial type; anatomical sampling site (e.g., mouth, gut, skin, liver); and methodological inconsistencies in sample collection and sequencing protocols [64,65,66].

Moreover, standardized bioinformatic pipelines are lacking, and the interpretation of microbial data requires sophisticated statistical tools or machine learning algorithms. While the concept of a predictive microbial PMI model is appealing, current efforts are often restricted to pilot studies with small sample sizes and limited generalizability.

In conclusion, microbial succession holds significant promise as a PMI estimation tool, but its forensic application is currently limited by methodological variability, environmental sensitivity, and a lack of clinical standardization.

### 4.6. Molecular Approaches (RNA-, DNA-, and Protein-Based)

Molecular methods for PMI estimation have gained considerable attention in recent years, with a particular focus on postmortem RNA degradation, protein fragmentation, and DNA integrity as time-dependent markers. These approaches offer potential advantages over conventional methods due to their biological specificity and quantifiable degradation kinetics. However, they remain under development, with significant challenges regarding standardization, reproducibility, and applicability in real forensic settings.

RNA-based techniques primarily investigate messenger RNA (mRNA) transcript decay or the expression of time-sensitive genes across different tissues. Studies, such as those by Zhu et al. (2017) and Javan et al. (2020), suggest that certain gene expression profiles change in a temporally correlated manner postmortem, supporting the concept of ”molecular clocks” for PMI estimation, although variability remains across tissues and environmental conditions [17,49]. According to the systematic review by Cianci et al. (2024), RNA markers such as GAPDH and ACTB have been widely studied for postmortem interval estimation, but their degradation rates are influenced by multiple factors and are not consistently reproducible across studies [21]. Despite promising results, this approach is highly sensitive to temperature, pH, and sampling delays, making prompt collection and strict storage protocols essential. Moreover, inter-individual biological variability and environmental exposure introduce high variability, as highlighted by Singh et al. (2025) [3].

RNA-based techniques are especially valuable during the early postmortem period (a few hours to ~2–3 days), as mRNA degrades rapidly due to endogenous RNase activity. Techniques, like RT-qPCR and RNA sequencing, have shown that certain transcripts follow reproducible, tissue-specific decay profiles. Zhu et al. (2017) identified over 260 genes with PMI-correlated expression patterns in muscle and brain tissues using GTEx data [17].

RNA-based techniques have the following advantages: high sensitivity, minimal sample volume required, and tissue specificity.

However, they have the following limitations: RNA is highly unstable and degrades rapidly depending on temperature, pH, and handling conditions. No universally validated transcript panels are available and interstudy reproducibility remains limited.

The RNA integrity number (RIN) is increasingly used to assess RNA quality, yet its application is limited by postmortem variability, environmental effects, and a lack of inter-laboratory standardization [50,67].

Protein-based methods assess the progressive breakdown of structural and enzymatic proteins. Protein-based methods extend the PMI estimation window to days or even weeks postmortem. Certain structural and enzymatic proteins, such as troponin, actin, and tropomyosin, degrade in predictable patterns. Zissler et al. (2020) reviewed studies showing consistent postmortem decay in skeletal muscle proteins, proposing their use in future forensic practice [18].

Protein-based methods have the following advantages: greater molecular stability than RNA and compatibility with standard (gel electrophoresis) and advanced (mass spectrometry) techniques.

However, they have the following limitations: there are variable degradation kinetics across different proteins; external factors, like temperature, moisture, and microbial activity, influence results. These methods remain in the experimental phase and require validated reference databases.

As noted by Gerra et al. (2024), proteomic PMI estimation remains constrained by the need for large datasets and robust validation across multiple populations and death scenarios [50].

DNA-based approaches, though less extensively explored, have recently been reconsidered due to advances in molecular biology. Although less explored, DNA degradation has gained interest for later PMI stages.

DNA-based approaches have the following advantages: DNA remains stable longer than RNA and may be useful when other markers fail. A systematic review by Bhoyar et al. (2025) assessed the utility of DNA fragmentation patterns, such as comet assay results, in PMI estimation [30]. Their findings showed that genomic DNA degradation follows a gradual trend in postmortem tissues, but external factors, like humidity, trauma, or decomposition, can drastically affect results. Unlike RNA, DNA is more stable, which may extend its applicability to later PMIs; however, high inter-sample variability and a lack of standardized protocols limit forensic implementation.

However, they have the following limitations: the research is still preliminary; results are affected by environmental and intrinsic variables, and their forensic applicability is not yet established.

As Cianci et al. (2025) and Gerra et al. (2024) emphasized, no molecular marker has reached sufficient validation for routine casework [21,50].

Although still under development, molecular approaches show great potential for improving PMI estimation. By targeting biochemical changes at the RNA-, protein-, and DNA levels, these methods offer a higher level of specificity than traditional techniques. As standardization improves and new technologies are integrated, molecular tools may become reliable complements to conventional methods in forensic practice.

### 4.7. Imaging Techniques

Postmortem imaging techniques, including infrared thermography, PMCT, and MRI, have emerged as useful adjuncts for PMI estimation—particularly in cases where traditional autopsy is not feasible. These methods allow non-invasive visualization of internal decomposition features, such as gas accumulation, fluid redistribution, or tissue changes, which may correlate with time since death.

Thermal imaging, for example, enables early estimation by tracking the dissipation of body heat (algor mortis), but its utility is confined to the first hours postmortem and is highly sensitive to ambient temperature and body insulation. PMCT has shown promise for staging decomposition by quantifying gas accumulation in organs, with liver and cardiac gas volumes being the most studied variables [14]. However, decomposition rates vary substantially across individuals and environments, which limits the precision of time estimates derived from imaging.

Infrared thermography improves the accuracy of algor mortis assessments compared to conventional thermometers by generating continuous cooling curves for the early postmortem period [11]. PMCT and MRI are valuable for investigating the cause of death, and their potential for PMI estimation is under investigation; however, the lack of dissection limits their diagnostic capacity. These methods have also been applied to human skeletal remains [68]. Here, we can mention potential methods for more rapid and accurate estimation of PMI on human skeletal remains, such as near-infrared (NIR) spectroscopy [51].

A key limitation remains the lack of standardized quantitative models linking imaging findings to PMI. Most studies are observational or based on animal data, and the interpretation often requires expert radiological evaluation. As López-Lázaro and Castillo-Alonso (2024) point out, imaging techniques alone rarely provide specific PMI windows but can support other methods in integrated, multimodal assessments [58].

Despite these constraints, imaging technologies contribute valuable context in forensic analysis. As computational tools, such as radiomics and artificial intelligence, evolve, these methods may be refined to enhance objectivity and reproducibility in PMI estimation.

### 4.8. Omics Technologies

Omics technologies—encompassing proteomics, metabolomics, lipidomics, transcriptomics, and epigenomics—represent one of the most innovative directions in PMI estimation. These approaches allow for the large-scale analysis of biomolecules (proteins, metabolites, RNA, and lipids) in postmortem samples, aiming to identify complex molecular patterns that evolve in a time-dependent manner after death. Instead of relying on single biomarkers, omics methods use machine learning and statistical models to interpret multidimensional data and generate predictive PMI signatures.

In proteomics, postmortem degradation of muscle proteins, such as tropomyosin and desmin, has shown a promising temporal correlation, especially in early- and mid-PMI ranges. Studies, such as those by Zissler et al. (2020) and Pittner et al. (2016), have shown that certain proteolytic fragments exhibit time-dependent and partially reproducible degradation patterns, supporting their potential use for PMI estimation over several days [18,47]. According to Procopio et al. (2018), and Bonicelli et al. (2022), proteomic analyses have also been applied to bone and mummified tissues in advanced stages of decomposition, offering potential to extend PMI estimation into later PMIs [52,56].

Metabolomics has revealed specific patterns of metabolic changes, such as the accumulation or degradation of purines, short-chain fatty acids, or amino acids. Du et al. (2018), using rat muscle tissue, demonstrated that these metabolite profiles could be used to classify postmortem samples by PMI with high accuracy during the first 72 h [53].

More recently, multi-omics strategies that combine transcriptomic, proteomic, and metabolomic layers have been proposed to increase precision. Secco et al. (2025) illustrated this approach by integrating data from different tissues to improve the resolution of PMI estimation [20]. The combined analysis allowed the identification of molecular signatures not evident when studying a single omics layer, pointing towards a more holistic and reliable forensic tool.

Epigenomic approaches are also emerging, focusing on DNA methylation changes after death. According to Gerra et al. (2024), these methods could provide stable time-dependent epigenetic markers with potential forensic utility, although the field is still in early development [50].

Despite their high potential, omics-based PMI methods face substantial limitations. Most available studies were performed on animal models under controlled laboratory conditions, which may not fully replicate real-world human decomposition scenarios. Even studies involving human cadavers often suffer from small sample sizes and lack standardized collection and analysis protocols. The implementation of these methods in forensic practice is further hindered by the need for expensive instrumentation (e.g., LC-MS, NGS), bioinformatics infrastructure, and specialized personnel. Additionally, the variability between individuals, causes of death, environmental conditions, and tissue types remains a significant challenge.

Nonetheless, as sequencing and analytical technologies continue to evolve, and as more human validation studies emerge, omics methods are likely to contribute to the future development of probabilistic, multi-parameter PMI models with defined error margins and clinical applicability [20,21].

### 4.9. Limitations of the Reviewed Studies

The studies included in this review show significant heterogeneity in terms of species (human vs. animal), environmental conditions, tissues analyzed, and methodological protocols. This variability limits comparability and prevents meta-analysis.

Many recent approaches, such as microbial profiling, RNA/protein degradation, and omics techniques, lack standardized protocols and are still in the experimental phase, with limited validation on human cases.

A large proportion of studies are based on animal models or controlled environments, which do not fully reflect real forensic scenarios. Moreover, few methods cover long postmortem intervals (>10 days), and most are focused on early-to-intermediate timeframes.

Environmental factors (e.g., temperature, humidity) and individual variability (e.g., microbiome, cause of death) significantly influence outcomes. Reporting quality is also inconsistent, with limited information on confidence intervals and reproducibility.

Despite these limitations, the review aimed to present a comprehensive and balanced synthesis of current PMI estimation strategies.

Overall, while the reviewed literature illustrates significant methodological progress, standardization and validation remain essential for integrating any new method into forensic casework. While traditional approaches gained credibility through centuries of empirical use [69], emerging techniques, such as RNA/protein degradation analysis and microbial succession, require rigorous validation through controlled studies with known PMIs and standardized protocols.

### 4.10. Future Directions

Future research should prioritize the standardization of protocols for molecular and microbiological methods, with a focus on validation in human forensic cases under real-world conditions. The promising results of RNA, protein, and microbial analyses require replication on larger, diverse populations and in different environmental contexts.

There is also a growing need for integrated, multi-parameter models combining traditional and novel biomarkers. The application of machine learning and AI-based predictive algorithms may enhance PMI accuracy by processing complex, multidimensional datasets.

Developing multi-omics platforms—which integrate genomic, transcriptomic, proteomic, and metabolomic data—could offer robust, time-sensitive profiles. However, their forensic implementation depends on improving reproducibility, reducing costs, and building comprehensive databases.

Interdisciplinary collaboration between forensic scientists, pathologists, molecular biologists, and data scientists will be essential to translate experimental findings into court-admissible tools.

### 4.11. Final Remarks

This systematic review underscores the growing diversity of methods for estimating the PMI. While classical signs, such as algor, livor, and rigor mortis, remain useful in the early phase, their precision is limited. Newer techniques, such as vitreous chemistry, forensic entomology, microbial succession, RNA and protein degradation, imaging, and omics, have expanded the investigative framework.

However, no single approach currently ensures reliable PMI estimation across all timeframes and scenarios. Many emerging methods remain experimental, lacking validation on human cases or standard forensic conditions.

The future of PMI estimation lies in multidisciplinary integration, combining complementary methods under standardized protocols and evidence-based models. Such convergence holds the potential to transform PMI estimation into a more accurate, reproducible, and court-admissible process—bridging the gap between research and real-world forensic application.

## 5. Conclusions

This systematic review summarizes the main categories of methods currently used to estimate the PMI, from traditional thanatological signs to modern molecular and omics-based approaches. While conventional methods remain useful in the early postmortem phase, their accuracy declines rapidly with time. Advanced techniques, such as forensic entomology, microbial succession, RNA and protein degradation, and omics platforms, offer future perspectives for extending the PMI estimation window.

The most promising results come from combining multiple approaches, e.g., traditional signs with biochemical markers or entomological evidence with omics data, to improve reliability. However, significant limitations remain, including variability across individuals and environments, limited validation in real forensic cases, and a lack of standardized protocols.

Recent research is increasingly oriented towards multimodal and data-driven models, integrating molecular profiles with environmental data using machine learning tools. Future developments should focus on large-scale human studies, standardization, and the creation of validated predictive models that can be applied confidently in forensic casework.

## Data Availability

The data presented in this study are available in the article [Table 2].
